# Supplementary material for: Neuronal avalanches in temporal lobe epilepsy as a noninvasive diagnostic tool investigating large scale brain dynamics
Source: Sci Rep. 2024 Jun 18;14:14039. doi: 10.1038/s41598-024-64870-3 (PMC11189588; doi:10.1038/s41598-024-64870-3)
Supplement: Supplementary file 1 — Supplementary Information. [file 41598_2024_64870_MOESM1_ESM.docx]

**
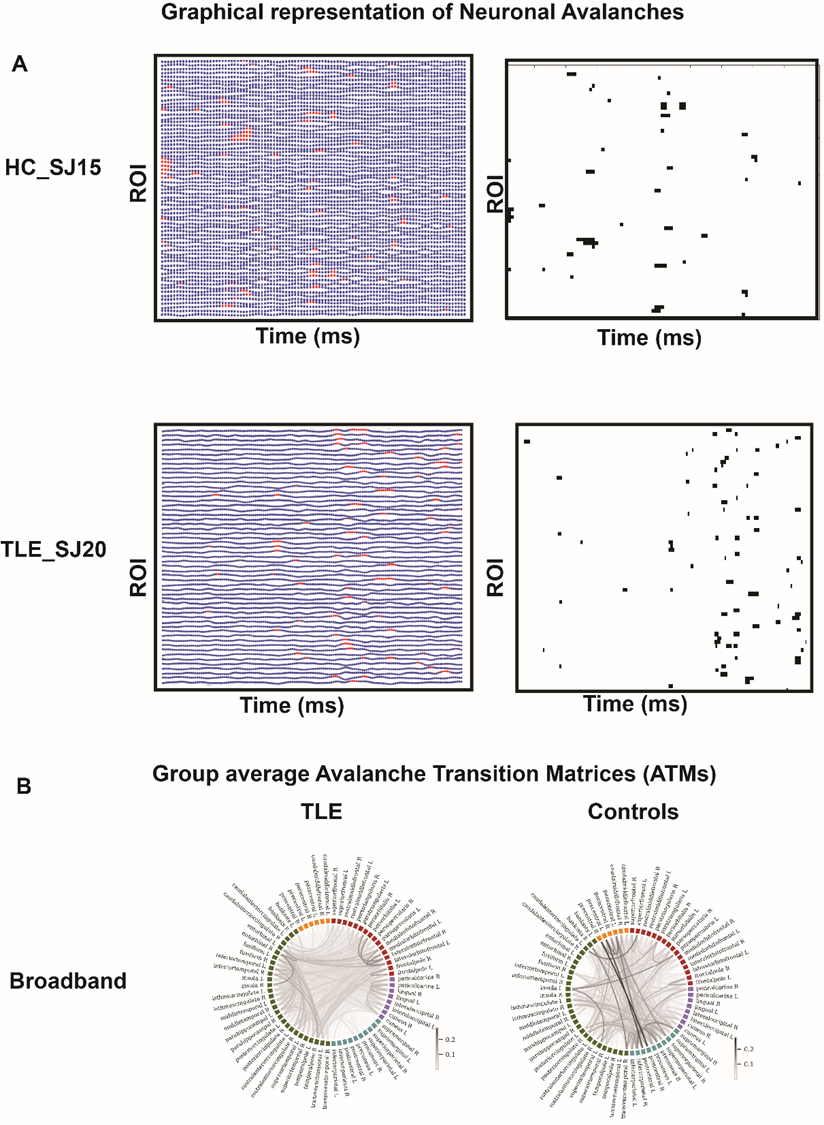
Supplementary Materials**

**Supplementary Figure 1. Graphical representation of neuronal avalanches and avalanche transition matrices.** The panel A of the present figure is a graphical representation of the neuronal avalanche computation for two randomly selected subjects in each of the groups (Temporal Lobe Epilepsy (TLE) and Controls). The blue dashed lines represent the time series of the brain parcel of 500 ms of recording. The red dots represent the time points in which the signal surpasses the threshold for avalanche extraction. The raster-plot on the right is an additional representation of the red parts of the signal, namely neuronal avalanches (NA). A NA starts when a region’s activity surpasses the threshold and it ends when all the activity is below the threshold. Panel B is a graphical representation of the average avalanche transition matrix (ATM) for the two groups.


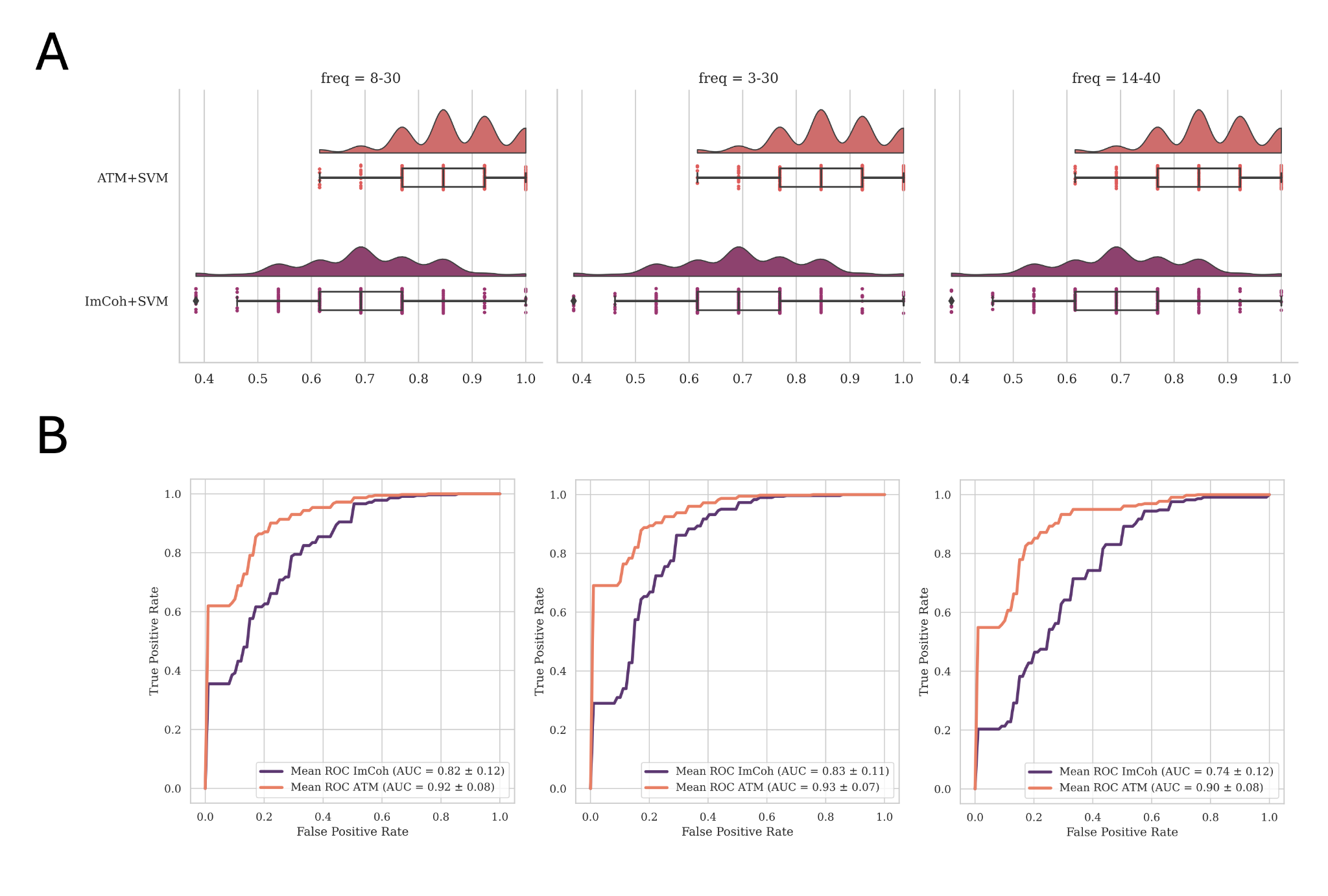


**Supplementary Figure 2. Accuracy and receiver operating characteristic curves.** Panel (**A)** of the present picture displays the full distribution of the classification accuracy of the support vector machine (SVM) model across the cross-validation splits, when trained with avalanche transition matrix (ATM, in salmon) and imaginary coherence (ImCoh, in purple). On the left it is reported the distribution of the accuracy classification using alpha-band filtered signal (8-30 Hz), in the middle theta-alpha-beta filtered signal (3-30 Hz), and on the right the beta-low-gamma filtered signal (14-40 Hz). Panel (**B)** shows the mean receiver operating characteristic (ROC) curves across cross-validation splits and the corresponding area under the curve (AUC), both for ATM (salmon line) and ImCoh (purple line).

|  | Alpha-Beta (8-30Hz)  mean ± SD | | Theta-Alpha-Beta (3-30Hz)  mean ± SD | | Beta-low gamma (14-40Hz)  mean ± SD | |
| --- | --- | --- | --- | --- | --- | --- |
|  | ImCoh+SVM | ATM+SVM | ImCoh+SVM | ATM+SVM | ImCoh+SVM | ATM+SVM |
| Accuracy | 0.69 ± 0.13 | **0.88 ± 0.10** | 0.76 ± 0.11 | **0.87 ± 0.09** | 0.61 ± 0.11 | **0.82 ± 0.08** |
| ROC AUC | 0.82 ± 0.12 | **0.92 ± 0.08** | 0.83 ± 0.11 | **0.93 ± 0.07** | 0.74 ± 0.12 | **0.90 ± 0.08** |
| F1-score | 0.62 ± 0.18 | **0.85 ± 0.13** | 0.72 ± 0.15 | **0.84 ± 0.10** | 0.43 ± 0.24 | **0.75 ± 0.14** |
| Precision | 0.66 ± 0.22 | **0.83 ± 0.17** | 0.72 ± 0.19 | **0.85 ± 0.15** | 0.49 ± 0.29 | **0.82 ± 0.17** |
| Sensitivity | 0.67 ± 0.24 | **0.90 ± 0.14** | 0.79 ± 0.19 | **0.86 ± 0.13** | 0.44 ± 0.29 | **0.73 ± 0.19** |
| Specificity | 0.74 ± 0.18 | **0.87 ± 0.12** | 0.77 ± 0.15 | **0.89 ± 0.11** | 0.77 ± 0.17 | **0.88 ± 0.10** |

**Supplementary Table 1. Classification performance**. The present table displays the indices of the classification performance when considering the model trained with avalanche transition matrix (ATM) or imaginary coherence (ImCoh) obtained respectively from the alpha-beta band (8-30Hz), the theta-alpha-beta band (3-30Hz) or the beta-low gamma band (14-40Hz) signal.


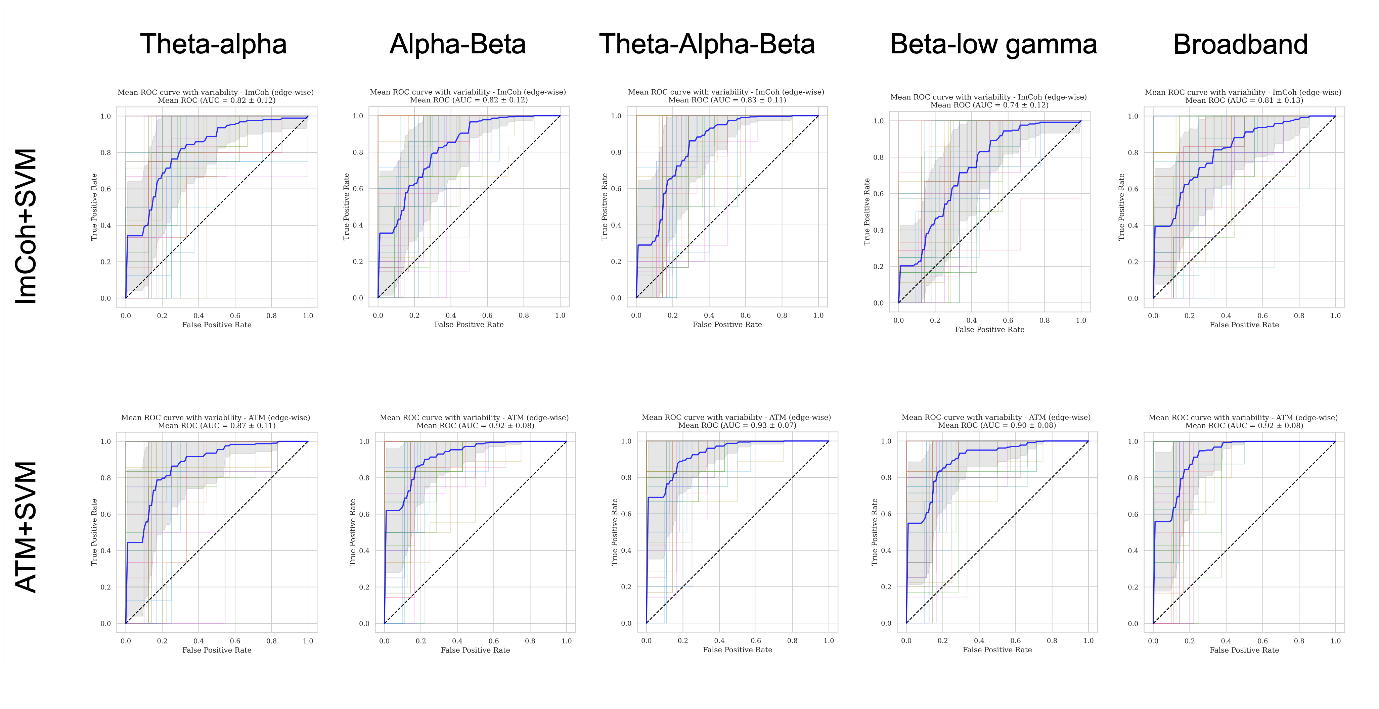


**Supplementary Figure 3. Receiver Operating Characteristic curves.** ROC Curves obtained with ImCoh+SVM and ATM+SVM within the broadband (3-40Hz) and the narrowband filtered signals (i.e., theta-alpha (3-14Hz); alpha-beta (14-30); theta-alpha-beta (3-30 Hz) and beta-gamma (30-40Hz)). Each figure represents the distribution of the ROC Curves across the splits and the blue line corresponds to the mean of the curves across splits.


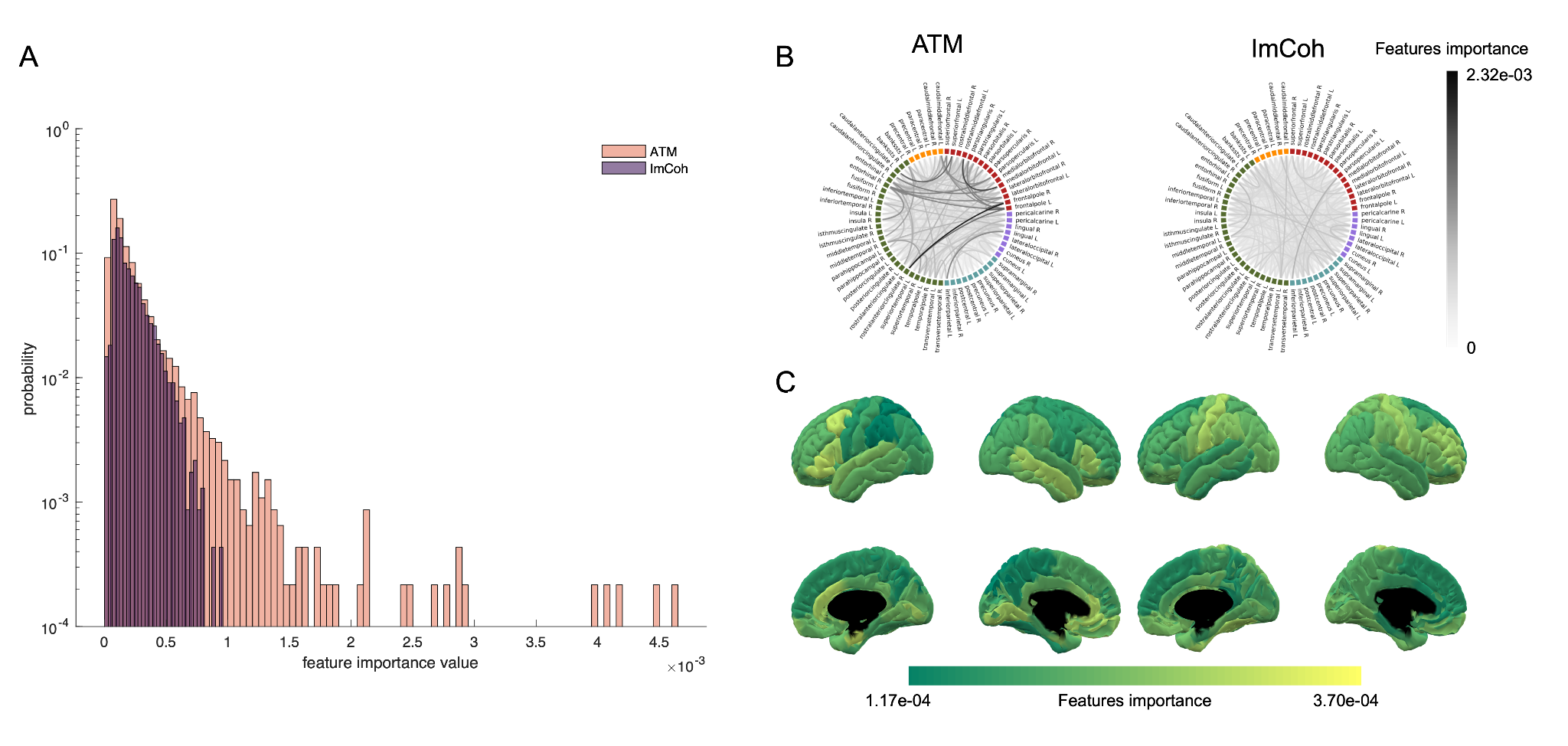


**Supplementary Figure 4. Feature importance for model interpretability (narrow band).** The present figure represents the relevance of the information used by the model to perform classification, namely the feature importance, both using avalanche transition matrix (ATM) and imaginary coherence (ImCoh). Panel A is a histogram of the probability of the feature importance value in ATM and ImCoh. The histogram shows a narrow distribution for ImCoh and broader distribution for ATM, suggesting that in ATM that certain edges drive the majority of information necessary for differentiation of the two groups. Panel B is the edge representation in a chord plot, showing the importance of each edge in the classification. Finally, panel C is the mean importance value of each edge of a specific brain region. This representation highlights which regions mainly impact in the classification.


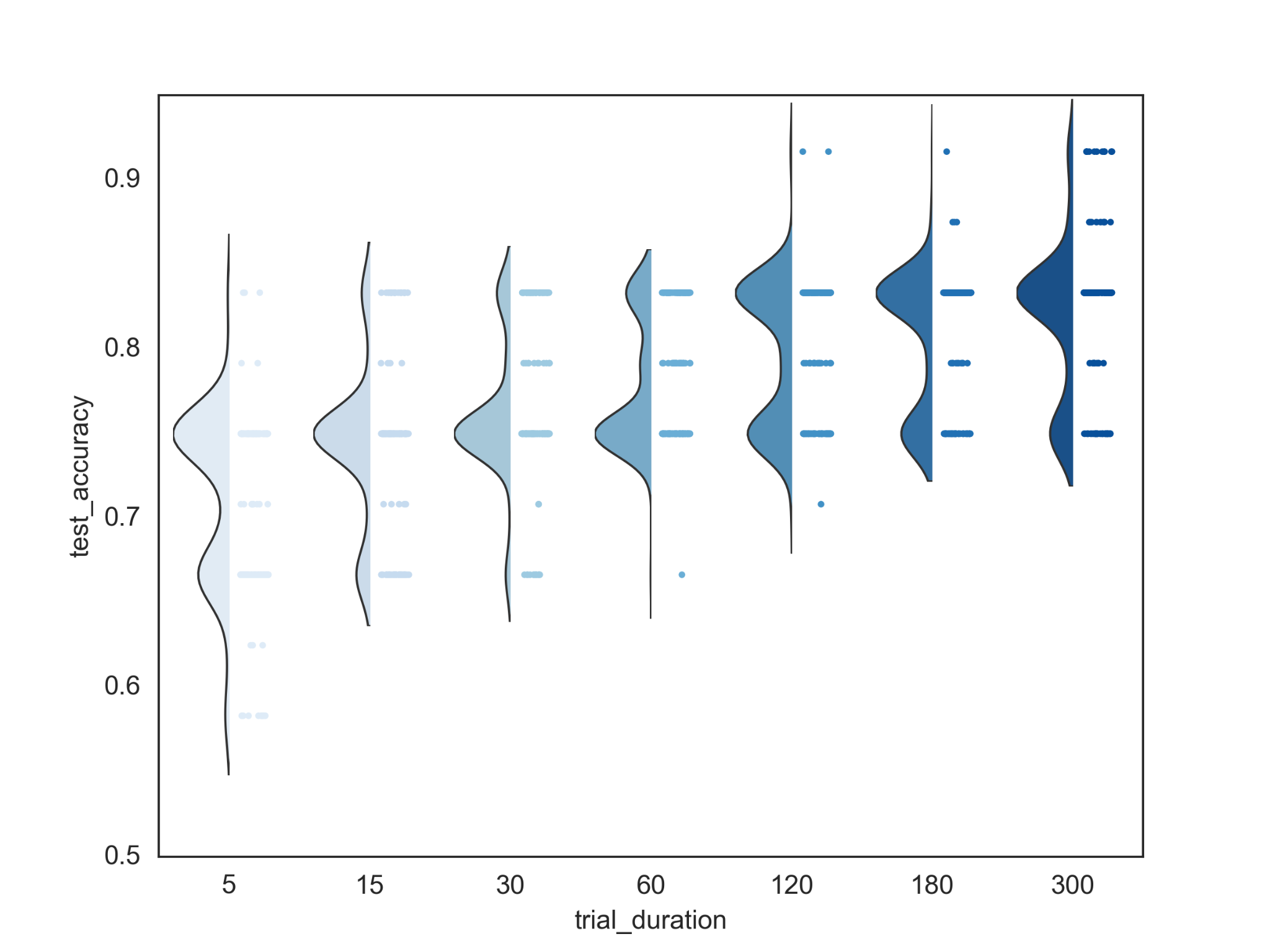


**Supplementary Figure 5 - Time dependence of classification accuracy - narrow band.** The picture shows the distribution across splits of the cross-validation based on the amount of signal used to compute avalanche transition matrix (ATM).
